# Effect of Multivitamins on the Color Stability of Dental Materials Used in Pediatric Dentistry: An In Vitro Study

**DOI:** 10.3390/polym16202948

**Published:** 2024-10-21

**Authors:** María Arregui, Josefina del Pilar Contreras Arellano, Ana Veloso Durán, Francisco Guinot Jimeno

**Affiliations:** 1 Dentistry Department, Faculty of Dentistry, Universitat Internacional de Catalunya, 08195 Sant Cugat del Vallès, Spain; mariaarregui@uic.es; 2 Pediatric Dentistry Department, Faculty of Dentistry, Universitat Internacional de Catalunya, 08195 Barcelona, Spain; jcontrerasa@uic.es (J.d.P.C.A.); fguinot@uic.es (F.G.J.); 3 Instituto de Investigación Sanitaria HM Hospitales, Madrid, Spain; 4 Service of Pediatric Dentistry, Hospital HM Nens, HM Hospitales, 08009 Barcelona, Spain

**Keywords:** color stability, nanohybrid composite, glass ionomer, Giomer

## Abstract

The longevity and acceptance of aesthetic dental materials are directly proportional to color stability. The aim of this study was to analyze the relationship between the use of multivitamins and the color stability of dental restorative materials. A total of 45 discs of nanohybrid composite, 45 of Reinforced Glass Ionomer (RGI), and 45 of Giomer were prepared. Subsequently, the samples were randomly divided into three solution groups (n = 15): Group 1—Sambucol Pediatric Syrup, Group 2—Hidropolivital Baby Drops, and Group 3—artificial saliva, which is preparation for patients with xerostomia. For 28 days, the specimens were immersed in 10 mL of each multivitamin for two minutes every 24 h. Color measurements were repeated on days 7, 14, 21, and 28. Statistical analysis was performed using the Jamovi software version 2.2.5, employing the Shapiro–Wilk test for normality and the Kruskal–Wallis test for non-parametric data. When comparing materials, statistically significant differences (*p* < 0.001) were observed between RGI and Giomer, and RGI and composite, but not between Giomer and composite (*p* = 0.716). The highest change was observed in RGI–Hidropolivital ΔE_00_ = 3.27 (2.38–4.59) and the least in composite–Sambucol ΔE_00_ = 0.72 (0.30–1.18). In conclusion, the exposure time and the multivitamin influence the color change of restorative materials.

## 1. Introduction

Physical appearance is often related to social acceptance and professional success; therefore, alterations to it can impact individuals’ quality of life [1]. This includes dental esthetics, which has become important for patient satisfaction in both adults and children [1,2]. It is important to consider that in pediatric dentistry, the demand for esthetics comes both from the parent and from the children [3,4]. A healthy smile is associated with physical attractiveness, and according to pediatric psychology, it is the means by which children begin to form interpersonal relationships and self-esteem, and affects the self-esteem of adults too [2,4,5]. As a result, research into the development of different dental materials that mimic both color and esthetics has increased in the few past years [2,3,4,6,7].

The most commonly used aesthetic materials in dental clinics are composites, glass ionomer (GI), and compomers [1,2,7,8,9]. All of these are used to restore posterior sectors, and composites and compomers can be used to modify dental anatomy, adjust misaligned teeth, close diastemas, and provide a tooth-like structure with acceptable aesthetic properties [8,9]. Composites are the most widely used materials in restorative dentistry (both in adults and children) but they are susceptible to staining, which can affect aesthetics [5,9]. Currently, there are different alternatives to these materials such as Bulk-Fill composites, dual-cure composites, ormocers, Reinforced Glass Ionomers (RGIs), Giomers and other Bioactive materials [6,10,11,12,13,14,15].

Within the different alternatives of restorative materials, Bulk-Fill composites and dual-cured composites are characterized by reducing polymerization shrinkage through different systems, as well as are less time-consuming and less techno-sensitive because they are used in higher increments versus the incremental technique [12,14]. An important factor to take into account is that in permanent dentition, they are capped with a conventional composite so they are not exposed to the oral environment [12,13,14], and esthetics is not relevant. However, Bulk-Fill composites in the primary dentition do not need to be covered by a conventional composite [12].

Meanwhile, Reinforced Glass Ionomers (RGIs), Giomers and Bioactive composites are considered an evolution of both composites and glass ionomers. On the one hand, RGIs are mainly characterized by a similar behavior to glass ionomers but incorporate composite components in their composition. This means that they retain the property of releasing and recharging fluoride to reduce the risk of new caries development but improve aesthetics [6,10,16]. On the other hand, Giomer is a hybrid material between composite and glass ionomer, so it represents a new type of composite that provides both protection against caries and erosion (glass ionomer), as well as mechanical and esthetical properties, and good polishing (composite), through the incorporation of reactive glass ionomer particles (S-PRG filler) in the composite matrix [6,11,15,17].

However, the longevity and acceptance of dental restorations made with these materials are directly proportional to the color stability of the material used, making this aspect one of the most important requirements when selecting a restorative material, as it will determine the long-term success or failure of the treatment [2,3,4,8,18,19,20].

In pediatrics, medicines in the form of syrups or liquid suspensions are regularly indicated to facilitate their intake, since swallowing capsules or tablets is often an uncomfortable and difficult process, especially in very young children [21,22]. The most used drugs are antibiotics, analgesics, antitussives and multivitamins, which are indicated to improve and protect the health of children [19]; however, some ingredients, such as colorant organic acid and sugars, can generate unwanted effects such as increasing the acidity of the mouth, favoring the cariogenic activity of oral bacteria [3,7,19,22]. Another way to administer drugs in pediatrics is inhalation, which was also seen to cause unwanted effects such as altering the color, texture, and roughness of restorative materials [9]. The latter promotes plaque accumulation and staining, thus affecting aesthetics and, therefore, the longevity of the treatment [9].

Multivitamins are dietary supplements that combine different vitamins to provide more nutrients to the human body and thus complement and strengthen the diet, and in such cases are necessary because the body cannot produce enough of them [23]. In children, multivitamins are prescribed in those cases where the body cannot produce enough of the necessary nutrients, which they contain, to support their growth and health [7]. They are prescribed for adults and children with nutrient-poor diets to meet special needs and improve overall health. It is important that they are taken with meals to enhance absorption and are not used to replace a balanced diet [18,21].

There is scarce literature on the effect of pediatric drugs, and dietary supplements, such as multivitamins, on the color stability of dental materials, but these medicines were reported to be an important cause of discoloration of restorations [1,19]. The literature describes multivitamin composition and the long treatment duration as possible factors associated with the color change caused by multivitamins [1,2,7,8]. Hence, the aim of the present study was to evaluate the color change of three restorative materials subjected to different multivitamins at different time points for 28 days. The null hypothesis was that the color change was clinically acceptable for all materials and solutions after a 28-day immersion time.

## 2. Materials and Methods

### 2.1. Study Design

This in vitro study was conducted with the approval of the University Ethics Research Committee in June 2023 (ODP-INVI-2023-02) in the Universitat Internacional de Catalunya (Barcelona, Spain) between December 2023 and March 2024.

The sample size calculation was performed using the Granmo calculator (version 7.12, Municipal Institute for Medical Research, Barcelona, Spain) accepting an alpha risk of 0.05% and a beta risk of 0.2% in a bilateral contrast. With a standard deviation of 0.5 and a minimum detectable difference of 0.4, the sample size was determined to be 13 specimens. As the dropout rate was not estimated in the sample size determination, it was decided that the final sampling rate would be 15 specimens per study group.

### 2.2. Specimen Preparation

The three materials tested in this study are described in Table 1, and the shade for all of them was A2. Forty-five disc-shaped specimens were made from each material using a metal mold with a diameter of 10 ± 0.1 mm and a thickness of 2 ± 0.1 mm. Each specimen was covered first with a sheet of polyester film and compressed using glass plates on the top and the bottom. Then, the specimens were light-cured on both sides for 20 s using a Demi Plus Kerr curing light unit (SDS Kerr Corp.; Middleton WI, USA; 1200 mW/cm^2^). A calibrated radiometer (Bluephase, Ivoclar VIvadent AG, Schaan, Liechtenstein) was used to verify the intensity of the light-curing unit. After the light-curing procedure, each specimen was polished with 320-grit silicon carbide paper to remove flash and irregularities to avoid roughness. Finally, they were demolded and stored in distilled water at 37 ± 1 °C for 24 h to complete the polymerization process in labeled containers.

### 2.3. Immersion Procedures

**Figure 1:**
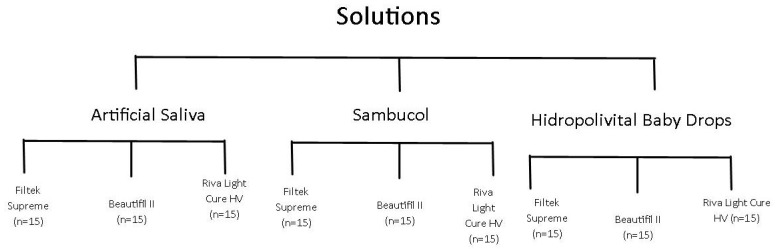
Distribution of materials in immersion solutions.

The multivitamin solutions and artificial saliva used in this study are listed in Table 2. The specimens (n = 15) for each material group (Figure 1) were immersed once a day for 2 min in 10 mL of each pediatric drug at 24 h intervals, during the waiting time the specimens were kept in artificial saliva at 37 ± 1 °C. The 2 min immersion every day was established in previous studies as time consumption of the drugs [7,20,23]. At the end of the immersion time, previously to reintroduce the specimens in artificial saliva, they were rinsed with distilled water and dry blotted with paper.

### 2.4. Color Measurement

Previous to all color measurements, all the specimens were rinsed with distilled water, passing through three beakers for 5 s each, and blotted dry with absorbent paper. The color measurements were performed at baseline (T0), 7 days (T1), 14 days (T2), 21 days (T3), and 28 days (T4).

Color coordinates L* (lightness axis), a* (red-green axis), and b* (yellow-blue axis) [21,22] were obtained using a dental spectrophotometer (SpectroShade M.H.T., S.p.a., Arbizzano di Negrar, Italy), which was calibrated with its own calibration device prior to each color session and after 15 measurements. All the measurements were performed by a single trained operator using a black background and with standardized D65 light illumination [24,25]. Three measurements were obtained for each specimen, and the average was recorded. The color change was calculated using the CIEDE2000 formula.

The CIEDE2000 formula measures the color difference by evaluating the effects of Lightness, Chroma, and Hue and the interaction of Chroma/Hue. This is performed with the following equation:

$$\Delta E_{00}\;\left(K_{L}:K_{C}:K_{H}\right)={\left[{\left(\frac{\Delta L^{\prime }}{K_{L}S_{L}}\right)}^{2}+{\left(\frac{\Delta C^{\prime }}{K_{C}S_{C}}\right)}^{2}+{\left(\frac{\Delta H^{\prime }}{K_{H}S_{H}}\right)}^{2}+R_{T}\left(\frac{\Delta C^{\prime }}{K_{C}S_{C}}\right)\left(\frac{\Delta H^{\prime }}{K_{H}S_{H}}\right)\right]}^{1/2}$$

where ΔL′, ΔC′, and ΔH′ are differences in Lightness, Chroma, and Hue, respectively. The weighting functions (SL, SC, SH) adjust the total color variation relative to the location in the L′, a′, b′ color space. The parametric factors (K_L_, K_C_, K_H_) are correction terms for the experimental conditions. Finally, RT corresponds to the interaction of differences between Chroma and Hue in the blue region [24,25,26].

There was evidence that the CIEDE2000 formula best adjusts to human perception in the color matching of specific shades in dentistry. The ∆E_00_ values were evaluated based on the specific perceptibility ∆E_00_ = 0.8 and acceptability threshold ∆E_00_ = 1.8 for polymeric materials in dentistry as reported in the literature [26,27,28].

### 2.5. Statistical Analysis

The statistical analysis was performed using the Jamovi statistical software (version 2.2.5). The Shapiro–Wilk and Levene’s tests were conducted to establish the normality and homogeneity of the data. Since, the data were non-parametric, the Kruskal–Wallis test (One way ANOVA for non-parametric data) was used to establish the significance level in the interactions between materials, times and solutions, and DSCF test was conducted for peer comparisons. A confidence level of 95% was established for all tests, and a significant difference was accepted when *p* < 0.05.

## 3. Results

The Kruskal–Wallis test observed that the color change presented statistically significant differences in relation to the material (*p* < 0.001), and the solution (*p* < 0.001), but not regarding time (*p* = 0.320).

**Figure 2:**
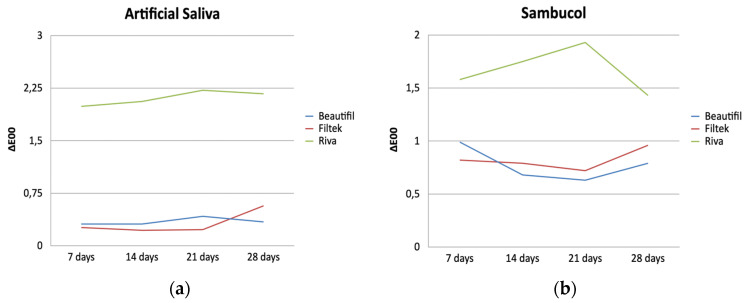

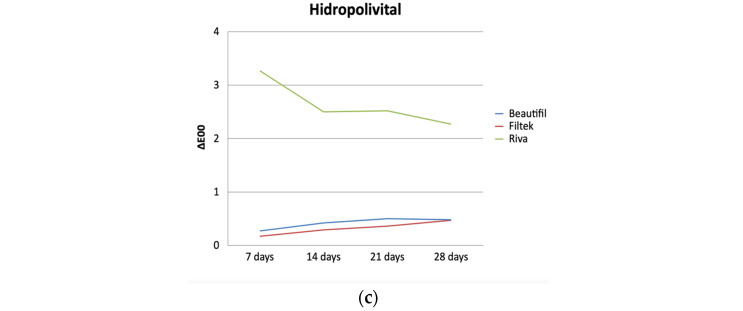
Color change of different filling materials in staining solution (**a**–**c**).

Table 3 summarizes the Mean ± Standard Deviation (SD), and Median (Minimum–Maximum) of the color change of the three tested materials after immersion in the different multivitamins and control group; Figure 2 presents the progression of the color change of each material and solutions at the corresponding different time points. The material with the lowest color change was different in each solution and time point. In artificial saliva, the composite (Filtek) exhibited the lowest ∆E_00_ for T1, T2 and T3; in Sambucol the composite showed the lowest ∆E_00_ for T1, and the Giomer (Beautifil) for T2, T3 and T4; and in Hidropolivital, composite exhibited the lowest ∆E_00_ for all time points. The material with the highest ∆E_00_ throughout all time points and solutions was reinforced glass ionomer (Riva).

The DSCF post hoc test in the pairwise comparison regarding the solutions revealed that statistically significant differences were found between artificial saliva and Sambucol (*p* < 0.001), and between Sambucol and Hidropolivital (*p* < 0.001), but no statistically significant differences were observed between artificial saliva and Hidropolivital (*p* = 0.058). The lowest ∆E_00_ values were obtained for artificial saliva (∆E_00_ = 0.43 (0.08–3.43)); intermediate values were obtained for Sambucol (∆E_00_ = 1.16 (0.30–3.47)), and the highest values were obtained for Hidropolivital (∆E_00_ = 1.20 (0.03–4.59)).

When comparing the different materials with the post hoc test, statistically significant differences were observed between Giomer and reinforced glass ionomer (*p* < 0.001), and composite and reinforced glass ionomer (RGI) (*p* < 0.001), but no statistically significant differences were observed between Giomer and composite (*p* = 0.716). The lowest ∆E_00_ values were shown by both Giomer (∆E_00_ = 0.52 (0.03–1.67)) and composite (∆E_00_ = 0.52 (0.08–1.91)), and the highest ∆E_00_ values were shown by reinforced glass ionomer (∆E_00_ = 2.25 (0.75–4.59)).

In this study, both composite and Giomer exhibited a color change that remained within the limits of perceptibility (ΔE_00_ = 0.8) and acceptability (ΔE_00_ = 1.8) at all time points (Table 3), while RGI was the only material that exceeded the limits of these thresholds, except for Sambucol multivitamin at T1, T2, and T4. RGI was the only material that showed in all immersion solutions a clinically unacceptable color change, as it exceeded the acceptability threshold.

## 4. Discussion

The objective of this study was to evaluate in vitro the impact of multivitamins on the color stability of three commonly used dental restorative materials in pediatric dentistry: composite, reinforced glass ionomer, and Giomer. The results reveal that the color stability of these materials is significantly affected by exposure to liquid multivitamins, while remaining within the acceptability threshold (∆E_00_ = 1.8), except for reinforced glass ionomer (RGI), which shows the highest susceptibility to color change above this limit. The null hypothesis was partially accepted since composite and Giomer comply with clinically acceptable color change in all solutions at 28 days, but RGIs did not present a clinically acceptable color change. This has important implications for clinical practice and the selection of restorative materials in children.

In pediatric dentistry, a clinically unacceptable color change of restorations is not only associated with esthetics and longevity of treatment, it can also generate parental concern and have a negative impact on children’s satisfaction and social interactions [1,3,4,7,8,18,19]. Therefore, replacement of the discolored restoration may become necessary in some clinical situations, involving increased expense and parental time, as well as the behavior management of children by the dentist [1,7,8,18]. This study aligns with previous research [1,2,8,18,20] indicating that both intrinsic material factors, such as the size and proportion of filler particles, hydrophilicity, polymerization depth and surface properties, and extrinsic factors, including exposure to colorants in foods, beverages, and medications, influence the discoloration of dental materials.

Of the three materials studied, the reinforced glass ionomer (RGI) showed the highest color change in the different multivitamins, which is similar to other studies [7,18,21]. This may be related to the following different factors: (1) the property of releasing fluoride; (2) lower content of filler particles; (3) its setting reaction, initially activated by light and subsequently an acid-base reaction will follow; and (4) the incorporation of HEMA in the organic matrix [7,18,21]. Different polyacrylic acids, such as itaconic acid or tartaric acid, are highly present depending on the composition of the reinforced glass ionomers. In the studied material, the presence of tartaric acid, which is a dicarboxylic acid, establishes a higher number of cross-links in the polymer chains, reducing spaces, thus decreasing water penetration and improving the water sorption resistance compared to conventional glass ionomers [29]. Therefore, the literature shows some evidence that the color of the glass changes due to the polymerization of this type of material, and furthermore, water sorption affects the content of resin and copolymers such as HEMA (a hydrophilic monomer) can cause a water intake over 80%, thus increasing the susceptibility to material staining [18,21]. Despite their anti-cariogenic and fluoride-releasing advantages, may not be the best choice in terms of color stability in the presence of liquid multivitamins [7,18].

In contrast, composite and Giomer show low levels of color change, even under the perceptibility threshold (∆E_00_ = 0.8) in artificial saliva and Hidropolivital groups, while remaining within the acceptability threshold (∆E_00_ = 1.8) in all multivitamins and control group. One reason for the lack of differences between composite and Giomer could be that both share a methacrylate-based composition, resulting in similar behavior.

In composites, the factors associated with maintaining their color stability could be the size and the amount of filler particles, organic matrix composition, the polymerization depth and the degree of conversion [7,10,18,21]. In Giomers, in addition to composite common factors, the color change may be associated with a high content of fluoride–borosilicate aluminum fillers obtained by the S-PRG technique, among other fillers, and their small size [3,4,10]. Different authors [3,4] suggest that materials that contain small particles have a smoother and more uniform surface, and, therefore, retain less pigment than rougher-surfaced materials.

The presence of S-PRG filler in Giomer is linked to the fact that by releasing ions; this type of filler has a buffering capacity reducing the acidic effect on the material itself [17]. Therefore, it could be assumed that the slight color change of this material is equally related to its composite-like composition, the small particle size, and its protective ability against the acidic agents of the S-PRG filler.

Similar studies [2,19,20] have indicated that pediatric medications in syrup form can cause significant discoloration in dental materials due to the presence of colorants and other additives. However, in this study, although clinically perceptible color differences were observed in some materials and solutions, no statistically significant differences were found over time, suggesting that discoloration stabilizes after an initial exposure period. This is consistent with the theory that most colorant absorption occurs quickly and stabilizes unless there is continuous exposure [24,25].

Another reason to justify the results of the present study is the change in the clinically perceptible and acceptable color change thresholds since there was a change in the formulas used in the measurements. Currently, their parameters have been greatly reduced, since the CIEDE2000 formula relates color change more closely to the human eye [26,27,28]. It must be taken into consideration that the perceptibility threshold refers to the magnitude of color difference that is visually detectable to the human eye, while the acceptability threshold corresponds to the magnitude of color difference that is considered clinically unacceptable [30].

According to these criteria, in the present study, only RGIs showed an unacceptable clinical color change. Further research would have to study whether the staining was superficial, which could be removed by polishing, or if the colorants penetrated sufficiently inside, which would make it necessary to replace the restoration.

Although these supplements are generally used for short periods of time, their repetitive use can have a significant effect on the properties of both tooth and restorative material, due to the erosive effect and low pH [3,7,19,21,23]. In this study, it was decided to standardize the time to 28 days, as this is the usual time for multivitamin consumption as in other studies [7,23].

In pediatric dentistry, dietary supplements such as multivitamins contain different minerals, vitamins, amino acids and plant-derived substances, which are generally dispensed in liquid form, in the form of syrups or drops to facilitate consumption in children [7,23]. The high viscosity of liquid medicines increases their tendency to stick to the surface of the tooth and the material for long periods of time and can cause different complications, including discoloration [19,21]. Another important factor is the solubility of some of the ingredients described above, because it is pH-dependent, so these drugs are formulated as acidic preparations to maintain chemical stability and optimize the dispersion and effectiveness of the medication [23]. Hence, multivitamins have a low pH that can cause softening or degradation of the restorative material, resulting in higher surface roughness, and, therefore, increased staining by pigment deposition on the surface [3,7,21,23]. The last factor of multivitamins with color change is the colorants used [7,23]. Aktas et al. [7] state that the difference in results observed in the literature may be related to the different formulations of the drugs studied in each study and the colorants used by the manufacturers. The multivitamins used in the present study were Hidropolivital with a yellow-orange coloration and Sambucol with a claret-red/purple coloration. Sambucol was the multivitamin that most affected the color change in composite and Giomer, while Hidropolivital caused a significant color change above the perceptibility threshold only in reinforced glass ionomer; this may be due to previously explained material-dependent factors.

These findings have several clinical implications. Firstly, pediatric dentists should be aware of the potential effects of multivitamins on the aesthetics of dental restorations. It is crucial to inform parents about the possibility of discoloration of reinforced glass ionomer restorations when administering multivitamins in syrup form. Additionally, it is recommended to consider selecting materials more resistant to discoloration, such as composites and Giomers, especially in situations where prolonged use of colored pediatric medications is expected.

A significant limitation of this study is its in vitro design, which cannot fully replicate the complex and dynamic oral conditions to which dental materials are exposed in the oral cavity. Factors such as pH variation, the presence of saliva, and interactions with foods and other agents cannot be perfectly simulated in a laboratory environment. Additionally, only two specific multivitamins were evaluated, limiting the generalization of findings to other similar products on the market. However, the results provide an important foundation for future clinical studies and for the selection of restorative materials in pediatric patients requiring frequent liquid medication. It would also be interesting to investigate the effect of different formulations of the same medications and the impact of specific oral hygiene practices on the color stability of dental materials.

Another limitation of the study was the inclusion of three restorative materials, Further research, should evaluate other materials such as bioactive composites, Bulk-Fill composites and dual-cured composites, taking into consideration that the latter two materials are not always exposed to the oral environment, as referred in the literature [12,14].

In future research, it should be evaluated the effect of multivitamins on mechanical properties such as flexural strength or microhardness, since these medications have low pH, in order to compare their effects with other research that has studied these properties subjected to substances with low pH [31,32].

## 5. Conclusions

The results of this study highlight the importance of considering color stability when selecting dental restorative materials for pediatric patients who consume liquid multivitamins. Reinforced glass ionomers showed the highest susceptibility to discoloration, while composites and Giomers demonstrated greater resistance to discoloration, making them preferable in situations where prolonged use of these medications is expected.

Pediatric dentists should consider these factors in clinical practice when planning restorative treatments to ensure long-lasting aesthetic results and when advising parents on the care of dental restorations in children.

## Figures and Tables

**Table 1:** Materials tested.

Brand Name	Material Type	Batch #LOT	Manufacturer	Composition *
Filtek Supreme XTE Universal	Nanihybrid Composite	100262213	3M ESPE, Congers, NY, USA	Matrix: Bis-GMA, UDMA, TEGDMA Fillers:72.5 wt%, 55.6 vol% silica, zirconia fillers
Beautifil II	Giomer	022352	Shofu, Kyoto, Japan	Matrix: Bis-GMA, TEGDMA, Fillers: multifunctional filler, S-PRG filler based on fluoboroaluminiosilicate glass
Riva Light Cure High Viscosity	Reinforced Glass Ionomer	1212859	SDI, Victoria, Australia	Liquid: polyacrlic acid, tartaric acid, HEMA. Powder: Fluoroaluminiosilicate glass

* Bis-GMA: Bisphenol A glycidyl methacrylate; UDMA: Urethane dimethacrylate; TEGDMA: Triethylene glycol dimethacrylate; HEMA: 2-hydroxyethyl methacrylate.

**Table 2:** Multivitamins and artificial saliva composition.

Solution	Composition	Manufacturer
Artificial saliva (control group)	Purified water, glycerol, lemon essence, nipagin sodium, saccharosse	Farmacia Xalabarder, Barcelona, Spain
Sambucol Kids	Glucose syrup, purified water, black elderberry juice (*Sambucus nigra*), Vitamin C (ascorbic acid), citric acid, potassium sorbate	Pharma Care Europe, West Sussex, UK
Hidropolivital Baby drops	Bottle: water, sorbitol, fructose, zinc gluconate, honey, pantothenic acid (vitamin B5), citric acid, E-433 emulgent, potassium sorbate, pyroxidine hydrochloride (vitamin B6), sodium fluoride. Plug: L-ascorbic acid (vitamin C), lactose, retinyl acetate (vitamin A), cholecalciferol (vitamin D), silicon dioxide, riboflavin 5′ sodium phosphate (vitamin B2), thiamine hydrochloride (vitamin B1), phylloquinone (vitamin K), cyanocobalamin (vitamin B12).	Menarini, Badalona, Spain

**Table 3:** Results of ∆E_00_—Mean ± SD—Median (Minimum–Maximum).

Solution	Material	T1		T2		T3		T4	
		Mean ± SD	Median (Min–Max)	Mean ± SD	Median (Min–Max)	Mean ± SD	Median (Min–Max)	Mean ± SD	Median (Min–Max)
**Artificial Saliva**	Beautifil	0.28 ± 0.15	0.31 (0.10–0.47)	0.31 ± 0.16	0.31 (0.08–0.52)	0.37 ± 0.20	0.42 (0.11–0.85)	0.33 ± 0.12	0.34 (0.11–0.59)
	Filtek	0.28 ± 0.13	0.26 (0.12–0.54)	0.30 ± 0.22	0.22 (0.08–0.99)	0.27 ± 0.15	0.23 (0.08–0.71)	0.50 ± 0.24	0.57 (0.12–0.96)
	Riva	2.03 ± 0.52	1.99 (1.14–3.24)	2.08 ± 0.53	2.06 (1.23–3.41)	2.32 ± 0.56	2.22 (1.39–3.42)	2.08 ± 0.56	2.17 (1.12–3.43)
**Sambucol**	Beautifil	0.93 ± 0.23	0.99 (0.55–1.23)	0.67 ± 0.22	0.68 (0.37–1.12)	0.63 ± 0.17	0.63 (0.30–1.03)	0.89 ± 0.29	0.79 (0.60–1.03)
	Filtek	0.82 ± 0.23	0.82 (0.49–1.36)	0.81 ± 0.263	0.79 (0.40–1.34)	0.70 ± 0.27	0.72 (0.30–1.18)	1.07 ± 0.41	0.96 (0.46–1.91)
	Riva	1.71 ± 0.46	1.58 (1.02–2.71)	1.92 ± 0.73	1.75 (1.01–3.28)	2.15 ± 0.80	1.93 (1.10–3.47)	1.63 ± 0.65	1.43 (0.75–3.24)
**Hidropoli-** **vital**	Beautifil	0.35 ± 0.25	0.27 (0.09–0.91)	0.45 ± 0.20	0.42 (0.09–0.85)	0.49 ± 0.28	0.50 (0.19–1.31)	0.55 ± 0.37	0.48 (0.03–1.67)
	Filtek	0.23 ± 0.11	0.17 (0.09–0.43)	0.31 ± 0.13	0.29 (0.14–0.64)	0.38 ± 0.16	0.36 (0.19–0.76)	0.59 ± 0.40	0.47 (0.21–1.55)
	Riva	3.19 ± 0.70	3.27 (2.38–4.59)	2.56 ± 0.79	2.50 (1.37–4.45)	2.78 ± 0.72	2.52 (1.94–4.59)	2.54 ± 0.86	2.27 (1.19–4.14)

## Data Availability

The data presented in this study are available upon request from the corresponding author.
